# A meta-analysis of baseline characteristics in trials on mite allergen avoidance in asthmatics: room for improvement

**DOI:** 10.1186/s13601-019-0306-3

**Published:** 2020-01-06

**Authors:** Frank E. van Boven, Nicolette W. de Jong, Gert-Jan Braunstahl, Roy Gerth van Wijk, Lidia R. Arends

**Affiliations:** 1 Department of Internal Medicine, Section of Allergology & Clinical Immunology, Erasmus Medical Center, P.O. Box 2040, 3000 CA Rotterdam, The Netherlands; 2 Department of Pulmonology, Sint Franciscus Vlietland Groep, P.O. Box 10900, 3004 BA Rotterdam, The Netherlands; 3 Department of Pulmonology, Erasmus Medical Center, Rotterdam, The Netherlands; 4 Department of Biostatistics, Erasmus Medical Center, Rotterdam, The Netherlands; 5 Department of Psychology, Education & Child Studies, Erasmus University Rotterdam, P.O. Box 1738, 3000 DR Rotterdam, The Netherlands

## Abstract

**Background:**

Evidence regarding the clinical effectiveness of mite allergen avoidance for the treatment of asthma is lacking. In previous meta-analyses on mite allergen control, the baseline data were not discussed in detail. This study updates and extends the existing Cochrane review by Gøtzsche and Johansen (Cochrane Database of Systematic Reviews, 2008, Art. No: CD001187), with a focus on baseline asthma outcomes and allergen exposures.

**Methods:**

We used the existing trials in the original Cochrane review and included newly published studies. The baseline data for the mite allergen load from the mattress, the standardized asthma symptom score (ASS), the forced expiratory volume in 1 s percentage of predicted (FEV_1_ %pred.), and the histamine provocative concentration causing a 20% drop in FEV_1_ (PC_20_) were extracted. First, the mean values of the outcomes were calculated. The influence of the mite allergen load was examined with a random-effect meta-regression using the Metafor package in R.

**Results:**

Forty-five trials were included; 39 trials reported strategies for concurrent bedroom interventions, and 6 trails reported strategies for air purification. The mite allergen load ranged from 0.44 to 24.83 μg/g dust, with a mean of 9.86 μg/g dust (95% CI 5.66 to 14.05 μg/g dust, I^2^ = 99.8%). All health outcomes showed considerable heterogeneity (standardized ASS mean: 0.13, 95% CI 0.08 to 0.18, I^2^ = 99.9%; FEV_1_ %pred. mean: 85.3%, 95% CI 80.5 to 90.1%, I^2^ = 95.8%; PC_20_ mean: 1.69 mg/mL, 95% CI 0.86 to 2.52 mg/mL, I^2^ = 95.6%). The covariate mite allergen load did not significantly influence health outcomes.

**Discussion:**

This meta-analysis shows that mite avoidance studies are characterized by the inclusion of patients with rather mild to moderate asthma and with varying and sometimes negligible levels of allergen exposure. Future studies should focus on patients with severe asthma and increased levels of allergen exposure.

## Introduction

House dust mite-allergic asthma is a prevalent disorder of the lower airways that affects hundreds of millions of people worldwide [1, 2]. The immediate allergic reaction to mites [3] suggests that controlling exposure to the antigen could be an appropriate first-line therapy for the treatment of mite-allergic asthma. However, guidelines and reviews provide ambiguous recommendations for mite allergen avoidance [4–6], reflecting a lack of consensus in this research field. This lack of consensus on the effectiveness of mite allergen avoidance is summarized by a Cochrane review [7], which was unable to demonstrate any clinical benefit of avoidance measures designed to reduce mite exposure in 55 trials. In addition to the substantial meta-analysis by Gøtzsche and Johansen [7], several other meta-analyses on mite allergen avoidance for the treatment of asthma report varying results for the effectiveness of avoidance [8–11]. The variation in the complex interventions as well as the heterogeneity of several study outcomes urges further exploration [12, 13].

The baseline data are a not well reported in the meta-analyses on the effectiveness of mite allergen control. These baseline characteristics provide attributes for evidence-based decision making in the daily practice of clinicians [14]. First, in the case of asthma, baseline characteristics are of particular interest because they reflect the level of asthma control and the asthma severity of the patient [15]. Studies still highlight the disparities between the asthma severity results between clinical trials and those reported from patient practice [16]. Treatable traits have been defined in severe asthma patients and may be associated with future exacerbation risk [17]. Second, baseline environmental aspects can influence the treatability of allergen-induced asthma [18]. Third, baseline characteristics provide statistical independence in the asthma outcomes of interest. This quantitative factor relates to the possible relationship between exposure and asthma outcomes; for example, in the paradigm of the bedding site introduced in the 1990s [19]. In such cases, the quantitative evaluation of the clinical effectiveness of the treatment of asthma in a meta-analysis differs from that of the traditional two-sample test [20]. These aspects demonstrate that baseline characteristics in a meta-analysis are important for the interpretation of the study results [21].

This study updates and extends the existing Cochrane review by Gøtzsche and Johansen [7], with a focus on baseline asthma outcomes and allergen exposures.

## Methods

### Searches and selections

The starting point for this protocol was the Cochrane review by Gøtzsche and Johansen [7]. This meta-analysis includes 55 trials. An updating search was performed in the EMBASE, Medline, and Cochrane databases (see Additional file 1: Appendix S1). The titles and/or abstracts of the retrieved updated studies were screened in Endnote by the first author to identify randomized trials that met the inclusion criteria. Searches and selections were checked by a second author (NWJ). We selected all trials by applying the following inclusion criteria; where possible, criteria derived from Gøtzsche and Johansen [7] was applied.

- The study was published in the English language.
- The study was a peer-review publication with full text (no abstracts).
- The study was a randomized controlled trial with blinding.
- The control included a placebo or no treatment (by Gøtzsche and Johansen [7]).
- The participants were physician-diagnosed with bronchial allergic asthma. These included participants who underwent a mite sensitization assessment with either a skin test or serum assay for specific IgE antibodies (by Gøtzsche and Johansen [7]). The asthma assessment included a history of asthma symptoms and a pulmonary function test.
- The intervention was designed to reduce the exposure to mite antigens in the home for the treatment of asthma (mono-trigger therapy by tertiary avoidance). This could include one of the following (by Gøtzsche and Johansen [7]):
  - Chemical (acaricides);
  - Physical (mattress covers, vacuum-cleaning, heating, ventilation, freezing, washing, air-filtration, and ionisers);
  - A combination of chemical and physical.

The flow chart of the updating search was made by use of the PRISMA diagram [22].

### Data extraction

The data extraction was elaborated by the first author (FvB); the extracted data included the study population, the type of intervention and control (the strategy of avoidance [13]), the study methodology (randomization and blinding), and outcomes. The outcomes included the main outcomes and the additional outcomes.

#### Main outcomes

- Mite allergen load from the mattress (μg/g dust).
- Asthma symptom score diaries (e.g. ASS/ACQ).
- Forced expiratory volume in 1 s percentage of predicted (%) (FEV_1_ %pred.)
- Histamine or methacholine concentration that causes a 20% reduction in the FEV_1_ (PC_20_).

#### Additional outcomes

- Medication usage (use of inhaled corticosteroids: yes or no).
- Type of patient (child or adult).
- Presence of co-sensitization.

Missing data were requested from the study authors. A second author (NWJ) validated the selections and the data extraction by the first author. Any ambiguities in the selections and the extractions were resolved by discussion.

The mite allergen load in trials was measured by the allergen content, the number of mites or the guanine content. A rapid colorimetric test such as the Acarex^®^ test can be used to measure the latter. Mite allergen exposure measured by Acarex^®^ or an equivalent test was excluded from the analysis; the Acarex^®^ test is poorly correlated with allergen content [23]. To estimate the allergen load from the number of mites in mattresses, the mean number of mites can be divided by a factor of 50. This ratio is adapted from a nonsensitization threshold for allergens and for mites [24]. However, confidence limits for this calculation are unknown. We therefore also excluded mite counts. The most reliable way to measure the allergen content is with a chemical assay; the Enzyme-Linked Immuno Sorbent Assay (ELISA). In an ELISA the house dust mite allergens in the dust extract binds to an antibody, and are consequently linked to an enzyme, producing a detectable signal correlating to the antigen concentration in the extract [25]. This assay has been the most acceptable assay since 1989 [26]. We limited the studies to those measuring the mass (μg/g dust) of the mite allergen loads in mattresses with ELISA. Early epidemiologic studies defined a threshold level of 10.0 μg mite allergen per gram of dust, above which asthmatic patients are in risk of asthma attacks [24]. Confidence boundaries were absent, reducing the threshold to a rule of thumb. Since then, there is a lack of papers on this threshold level, and thus never updated.

Questionnaires have been developed to measure asthma symptom scores and the adequacy of asthma control, regarding shortness of breath, wheeze, woken by asthma, severity of asthma in the morning, limiting activities because of asthma, use of a short-acting bronchodilator [27]. A limitation of the ASSs is that are no validated cut-off points indicating severity or level of control. In the validated questionnaire by Juniper, an ACQ of 1.50 (maximum 6) relates to inadequately controlled asthma, [28], corresponding to a standardized cut-point of 0.25. The FEV_1_ measures the obstruction in the airways during a forced expiratory flower using a spirometer [15]. An FEV_1_ %pred. of 50 to 79% refers to moderate airflow obstruction, and < 50% to (very) severe obstruction [29]. In a standardized bronchoprovocation test, the dose histamine or methacholine is determined causing a 20% fall in FEV_1_, PC_20_ or PD_20_ [30]. A PC_20_ < 1 mg/mL is considered a severe airway hyper responsiveness, and > 8 mg/mL as being a normal responsiveness [31].

The analysis was limited to the main health outcomes with the most reported units. In the case of the ASS, we a priori standardized (SMN) the mean (MN) score by dividing it by the maximum number of the score (MAX). The variance was standardized in the same way (SD_standardized_^2^ = SD_extracted_^2^/(MAX^2^ * number of patients)).

### Risk of bias assessment

Gøtzsche and Johansen [7] judged the adequacy of the allocation concealment according to the Cochrane guidelines [32]. Their assessment was not included in the data synthesis. The trials selected for the updated analysis were assessed similarly for the risk of bias by the first author (FvB) using the Cochrane checklist [32]. A second author (NWJ) validated the assessment by the first author. Any ambiguities in the assessed risk of bias were resolved by discussion. We also did not include the assessments in the data synthesis, as we did not hypothesize that the risk of bias or the quality of trials would affect the baseline characteristics.

### Statistical and sensitivity analyses

The effect size was set as the mean for the physiological outcomes. The ASSs were standardized. First, the overall effect of the three health outcomes was estimated using a random-effects meta-analysis. Additionally, the I^2^ value was calculated to examine heterogeneity in the outcomes. A random-effect meta-regression and subgroups were introduced for all medical outcomes showing at least moderate heterogeneity. Covariates and subgroups of interest included the mite allergen load from the mattress at baseline and possible confounding by the use of inhaled corticosteroids, the type of patient (child/adult), and the presence of co-sensitization. Random-effects meta-regressions and subgroups were tested for a preferred minimum of ten trials [32]. Another sensitivity analysis yielded the exclusion of possible outliers as well as the results of the updated reference search. All calculations were performed with the Metafor 2.0.0 package in R 3.5.3. [33, 34]. The level of significance was set to α = 0.05.

## Results

### Selection of references

**Fig. 1:**
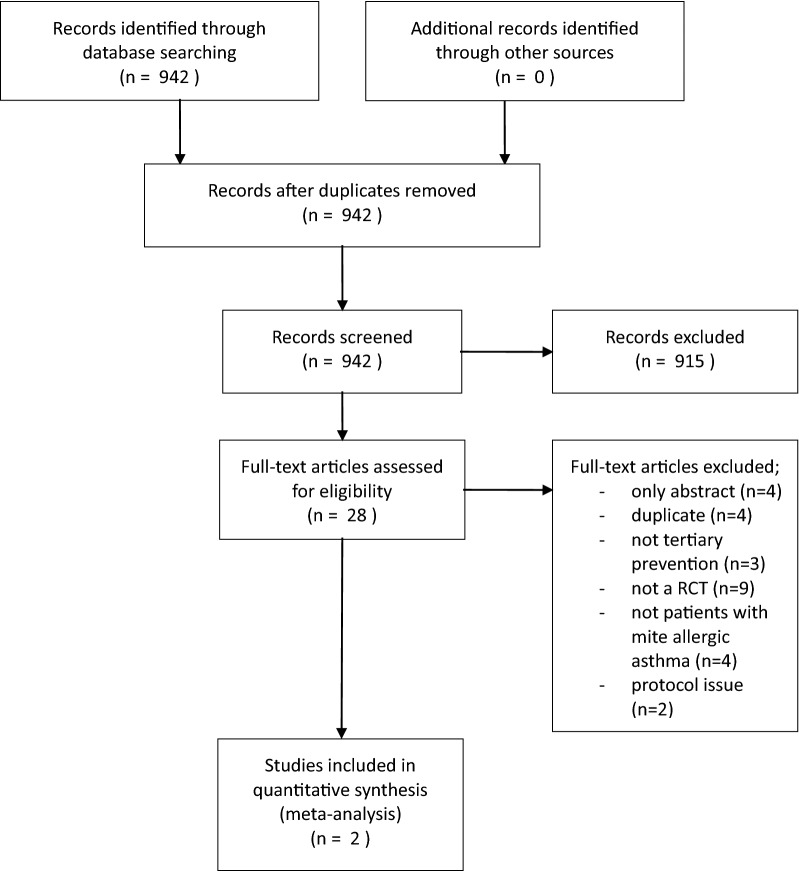
Flow chart of the updating literature search and selection of studies

The selection and inclusion of studies resulted in two groups of publications. The first group included the trials from the Gøtzsche and Johansen [7] analysis (fifty-five trials published until July 2011 [35–89]). We excluded twelve of these trials for being only abstracts, being published in a non-English language, not reporting data on the treatment of mite-allergic asthma, or containing non-usable data (outcomes not of prior interest; incomplete data) [35–45, 87]. One of the excluded trials was a large trial by Woodcock et al. [87], which dominated the meta-analysis by Gøtzsche and Johansen (weight > 40%). Woodcock et al. [87] reported incomplete data in the subset of the mite load as well as the ASS. Further, the research team did not report the FEV_1_ or the PC_20_ data. The remaining forty-three trials were included for data extraction. The second group included studies identified in our updated search starting in July 2011 (Fig. 1). We found a total of 942 titles and abstracts. Nine hundred and fifteen titles were excluded for not reporting a randomized blinded trial on the effectiveness of tertiary mite allergen avoidance. Twenty-eight potentially relevant titles were selected for inclusion [90–117]. Twenty-six full-text articles were excluded for not meeting our inclusion criteria (see Additional file 1: Appendix S1). Two full-text articles were included in the analysis [97, 115]. Finally, forty-five full-text articles were included in the analysis.

### Description of the included trials

Thirty-nine trials reported avoidance using concurrent bedroom intervention strategies, and six trials reported air purification strategies. In twenty-five trials (56%), patients used inhaled corticosteroids at baseline. Twenty-one trials reported on the treatment of children with allergic asthma, the other twenty-four reported on the treatment of adults; some trials included both children and adults. In nineteen trials, co-sensitization at baseline was reported. Gøtzsche and Johansen [7] previously reported that eight of the included trials had a low risk of bias. Seven trials were judged to have a high risk of bias. The bias in the remaining twenty-eight trials was deemed unclear by Gøtzsche and Johansen [7]. We judged the trial by El-Ghitany and El-Salam [97] to have an unclear risk of bias (no information on concealment was included). The trial by Murray et al. [115] was judged to have a low risk of bias (use of a computer-based minimization procedure).

### Mean characteristics at baseline

**Fig. 2:**
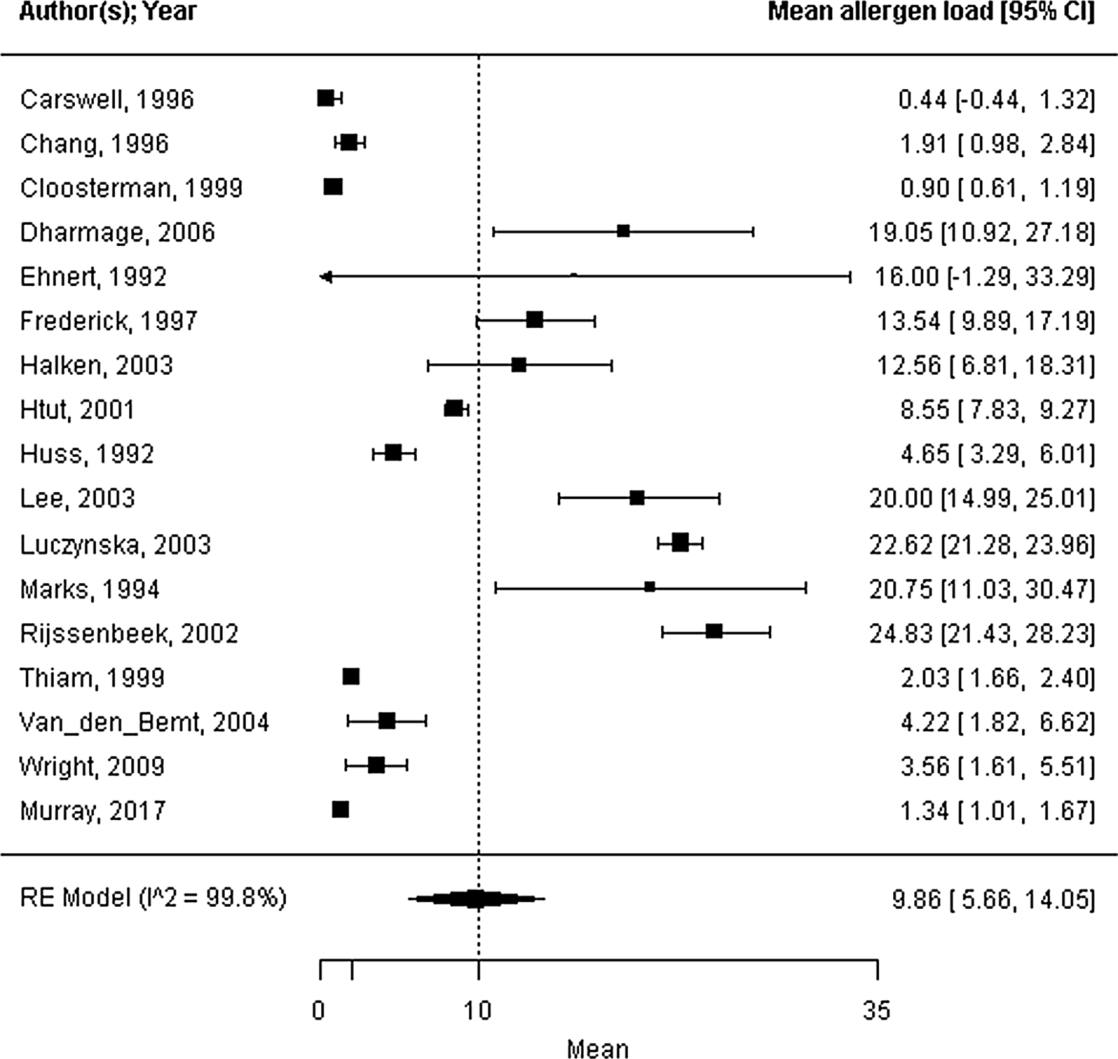
Forest plot of the mite allergen load of the mattress at baseline

**Fig. 3:**
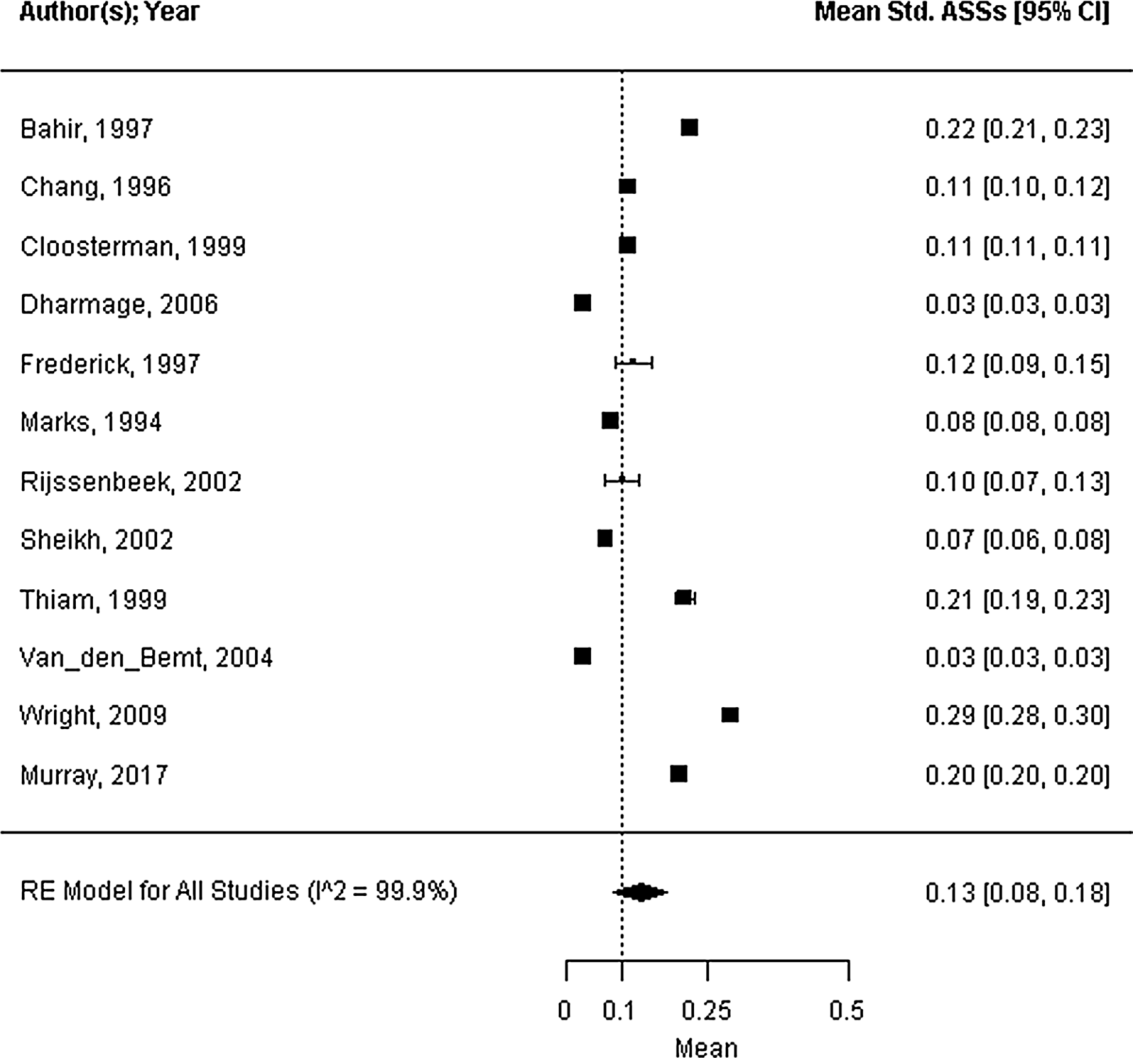
Forest plot of the standardized asthma symptom scores at baseline

**Fig. 4:**
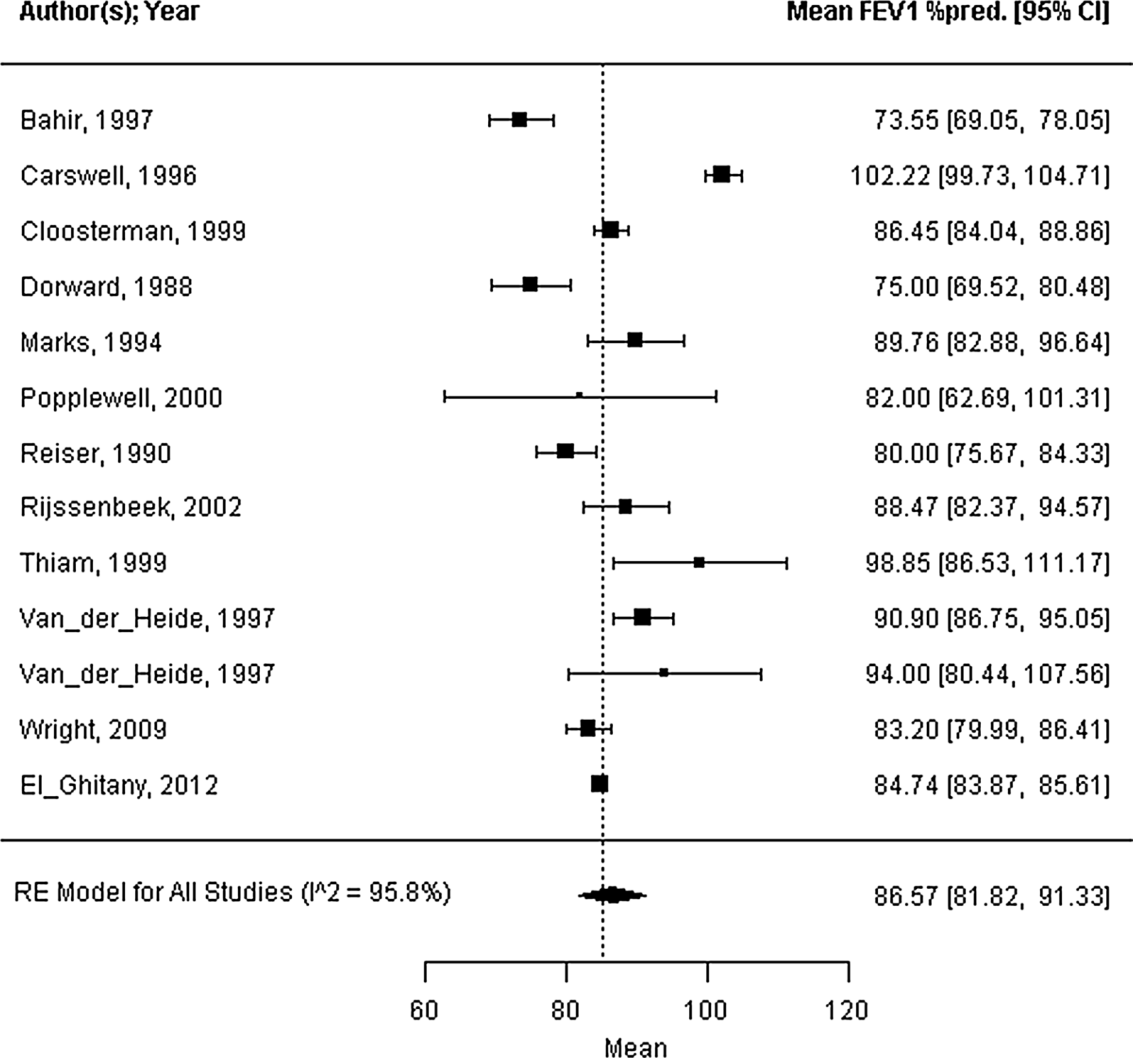
Forest plot of the FEV_1_ percentage of predicted at baseline

**Fig. 5:**
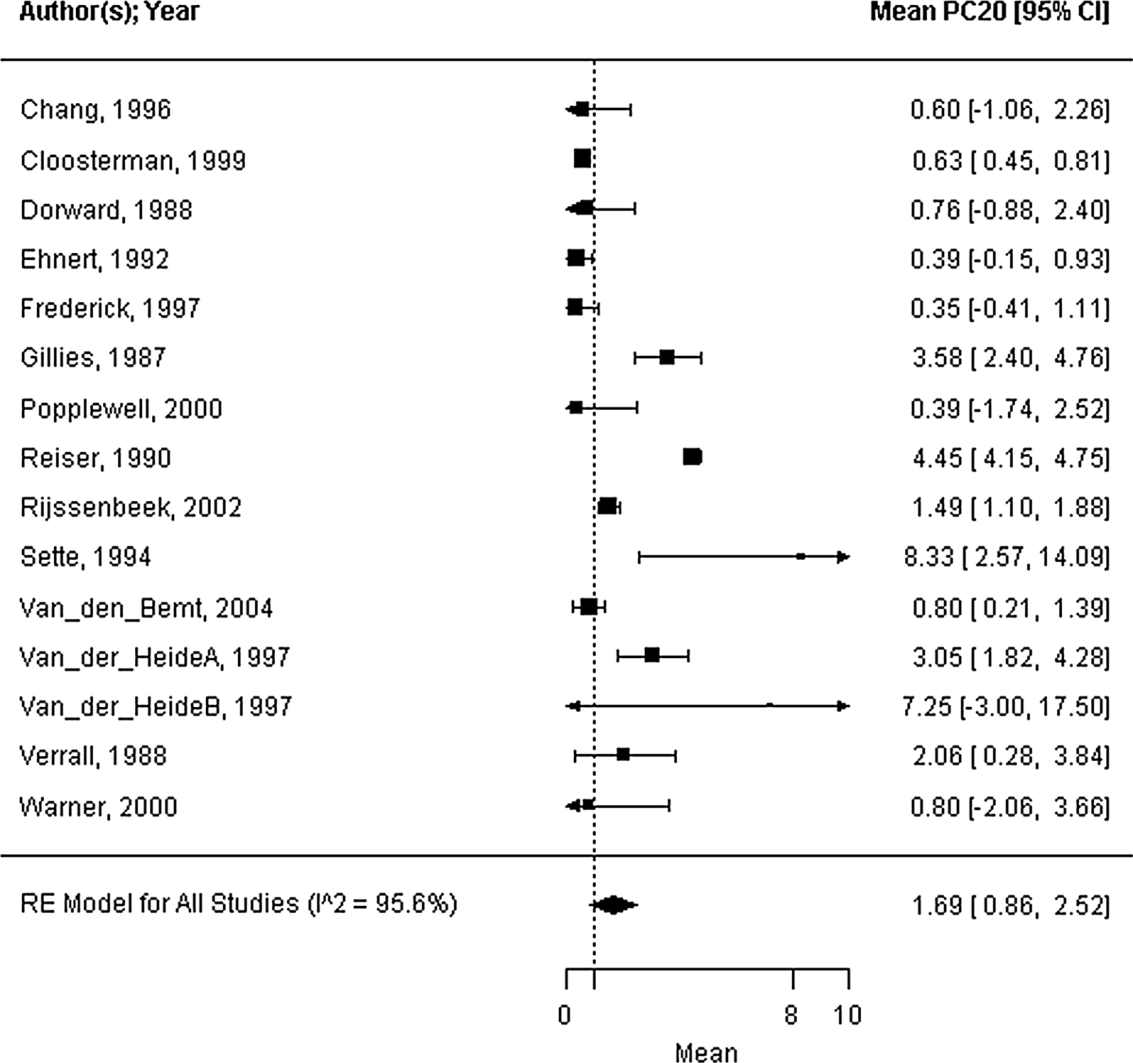
Forest plot of the PC_20_ at baseline

Seventeen of the forty-five trials reported on the mite allergen load from the mattress at baseline, as measured by ELISA (mean 9.86 μg/g dust; 95% CI 5.66 to 14.05 μg/g dust; range 0.44 to 24.83 μg/g dust; n = 1066; I^2^ = 99.8%; Fig. 2). The standardized ASSs at baseline were reported in twelve trials with high heterogeneity (standardized symptom score = 0.13; 95% CI 0.08 to 0.18; range: 0.03 to 0.29; n = 703; I^2^ = 99.9%; Fig. 3). Sixteen studies reported the outcome FEV_1_ %pred. by measuring the percentage predicted value (FEV_1_ %pred. = 85.3%; 95% CI 80.5 to 90.1%; range 68.5 to 102.2%; n = 816; I^2^ = 95.8%; Fig. 4). Fifteen trials reported PC_20_ values at baseline, expressed as mg/mL. The mean PC_20_ was 1.69 mg/mL (95% CI 0.86 to 2.52 mg/mL; n = 599; I^2^ = 95.6%, Fig. 5).

### Dependence, subgroups and sensitivity analysis

The covariate mite allergen load at baseline did not significantly influence the health outcomes (standardized ASSs: P = 0.13; FEV_1_ %pred.: P = 0.81; PC_20_: P = 0.75, see Additional file 1: Appendix S1). We calculated the FEV_1_ %pred. in the adult subgroup (FEV_1_ %pred.; adults = 84.2%, 95% CI 79.2 to 89.2%; 11 trials). All other subgroups included less than ten trials. Finally, the random-effects models for the health outcomes were unaltered when excluding the updated trials (symptom score 0.12; FEV_1_ %pred.: 85.4%; PC_20_: 1.69 mg/mL).

## Discussion

This study contributes to the existing Cochrane review by Gøtzsche and Johansen [7] by generating hypotheses on the characteristics of asthma outcomes according to baseline data as well as possible dependencies for asthma outcomes. We observed considerable heterogeneity in the mite allergen load in the mattresses (17 trials), the standardized ASSs (12 trials), the FEV_1_ %pred. values (16 trials), and the PC_20_ values (15 trials). We judged the mean mite allergen load from the mattress at baseline to be moderate (9.86 μg/g dust). Overall, the standardized ASSs and the percentage predicted FEV_1_ %pred. suggested a mild to moderate disease. The PC_20_ at baseline predominantly indicated moderate to severe airway hyperresponsiveness according to the definition by Cockroft [31]. We did not observe a relationship between the mite allergen load from the mattress at baseline and health outcomes. The number of trials available did not allow for comparisons between the child and adult subgroups, the inhaled corticosteroid use or no use subgroups, or the presence or absence of co-sensitization subgroups.

In this study, we observed several factors related to the three attributes of prior interest. The first attribute was asthma severity. We observed a mild to moderate magnitude of asthma severity at baseline. We were, however, limited in our evaluation of asthma severity by the absence of appropriate instruments to assess asthma control [27, 118] and the asthma-related quality of life [119]. Compatible with the situation of pharmacological treatments [16], it remains unknown whether the results found by Gøtzsche and Johansen [7] are generalizable to patients with uncontrolled asthma. In one trial [55], we extracted a median symptom score at daytime of zero for the treatment group. Since the score was already zero at baseline, it was probably clear that there would be no clinical benefit observed in this subset. The asthma outcomes showed more notable levels, such as a FEV_1_ %pred. above 100%, as reported by Carswell et al. [51]. The moderate asthma status at baseline was possibly related to the use of inhaled corticosteroids, as reported in more than half of the included trials (56%). However, the number of trials available did not allow for testing this hypothesis.

A second attribute is the magnitude of the exposure at baseline, which relates to the environmental treatability. In four of the included trials [51–53, 115], we observed that the mean mite allergen load from the mattress at baseline was quite low (range 0.44 to 1.91 μg/g dust). Only one of these four trials included an evaluation of the treatability of mite allergen exposure at baseline in their methods [52]. Environmentally, whether such low values of exposure are considered treatable remains a question. An exposure level of 0.44 μg/g dust is quite similar to the exposure level observed in the “low-allergen” region of Davos in the European Alps (approximately 0.02 to 0.2 μg/g dust; assessed from [120]). In addition, Pingitore and Pinter [121] noted that in many trials, there was no success in reducing the mite allergen load. Overall, it seems that multiple clinical trials on avoidance paid little attention to the environmental issue of the treatability of the exposure.

Furthermore, the attribute of dependence was of interest in this study. None of the medical baseline data could be related to mite allergen exposure from the mattress. This indicates that from a meta-viewpoint, at baseline, there was no clinical potential for reducing the mite allergen load in the bedding.

As far as we know, this is the first systematic review of baseline characteristics in trials on mite allergen avoidance for the treatment of asthma. This study was executed a priori to generate hypotheses for a new meta-analysis on the treatment of mite-allergic asthma by environmental control. Generating hypotheses to define a protocol for a meta-analysis prevents misleading conclusions [32]. We could not generate a hypothesis on a possible relationship with asthma outcomes, particularly considering the mite allergen exposure covariate. The mite allergen load from the mattress covariate was limited to the data obtained from ELISA. This limitation can be considered a rigorous selection factor to prevent bias in this covariate of prior interest. It is possible that some of the covariates we used were still unrefined. For instance, the covariate co-sensitization was introduced as a binary value (presence yes or no); we believe the next step is to introduce the number of co-sensitizations as an ordinal covariate.

The main limitation of this study was that we had to exclude the large trial by Woodcock et al. [87] because their data was not usable data for the purpose of this study. Woodcock et al. did probably not include patients with uncontrolled asthma. Their publication included only adult patients with asthma who were undergoing routine management with inhaled corticosteroids in primary care. Though not a limitation, another large trial also worth noting is the recently published study by Murray et al. [115]. Murray et al. found that only the use of single covers prevented asthma exacerbations in the hospital setting. In a post hoc analysis, Murray et al. reported that relatively younger children (P = 0.006), those mono-sensitized to mites (P = 0.04), those with severe asthma (P = 0.03), and those not exposed to smoking (P = 0.02) explained the reduced number of hospital admissions in the 123 participants. No information was presented on the selection of significant covariates or on the power of the calculations. Possibly, the results by Murray et al. [115] are explained by a more severe asthma status at baseline than those in the participants in the trials included by Gøtzsche and Johansen [7].

The baseline characteristics in a meta-analysis have been the subject of methodological studies, emphasizing the careful consideration of this topic in the definition of the protocol [21, 122]. Advanced statistical methods to evaluate underlying risk have been developed for cases in which the baseline characteristics or the severity of the disease among the participants varies [123]. The definition of the types of participants is considered a key factor in reviews [32]. A positive example of the explicit (a priori) consideration of baseline characteristics was demonstrated in the Cochrane review on the treatment of asthma by sublingual immunotherapy [124]. In contrast, the current meta-analyses on the treatment of asthma using avoidance were commonly characterized by no baseline characteristic reporting [7–11]. Gøtzsche and Johansen [7] stated that adjusting for baseline differences would risk biasing the review, “since investigators are inclined to show baseline differences and adjust for them when this procedure favours the experimental treatment”. By limiting their meta-analysis to the changes and final values, Gøtzsche and Johansen [7] did not account for the types of participants they reviewed. Other Cochrane reviews on the treatment of asthma or rhinitis by mite allergen avoidance [125, 126], recognized for their rigorous methodology, do not account for the types of participants, as they did not describe their baseline characteristics. This suggests that there is room for improvement in the multiple Cochrane reviews and other meta-analyses on avoidance.

In conclusion, this systematic review demonstrates that many previous mite avoidance studies are characterized by the inclusion of patients with rather mild to moderate asthma and with varying and sometimes negligible levels of allergen exposure. Most likely, the use of asthma medication modified the baseline asthma outcomes in these studies, leaving less room to improve. In future studies, we suggest focusing on patients with partially controlled or uncontrolled asthma and assessing asthma control with the appropriate instruments [27, 118, 119]. Moreover, to test the efficacy of allergen avoidance, sufficient mite exposure at baseline should be present. In the absence of an evidence-based threshold level, we suggest the provisional use of the formerly defined rule of thumb that suggests that 10.0 μg mite allergen per gram of dust is relevant to asthma symptoms [19].

## Supplementary information

**Additional file 1.** Supplemental information on the keywords of the reference search; list of included and excluded studies in the updated search; the number of trials available per subgroup; figures of the health outcomes as a function of the allergen exposure.

## Data Availability

The datasets used and/or analysed during the current study are available from the corresponding author on reasonable request.
