# Supplementary material for: A meta-analysis of baseline characteristics in trials on mite allergen avoidance in asthmatics: room for improvement
Source: Clin Transl Allergy. 2020 Jan 6;10:2. doi: 10.1186/s13601-019-0306-3 (PMC6943957; doi:10.1186/s13601-019-0306-3)
Supplement: Supplementary file 1 — Additional file 1. Supplemental information on the keywords of the reference search; list of included and excluded studies in the updated search; the number of trials available per subgroup; figures of the health outcomes as a function of the allergen exposure. [file 13601_2019_306_MOESM1_ESM.pdf]

Appendix S1

Reference search; keywords for embase.com

('Pyroglyphidae'/exp OR 'mite'/de OR 'Acari'/de OR 'house dust'/de OR 'house dust allergen'/de OR 'mite infestation'/de OR 'house dust allergy'/de OR 'dust exposure'/de OR (Dermatophagoid* OR mite OR mites OR 'D farinae' OR 'd pteronyssinus' OR Pyroglyphid* OR Euroglyph* OR 'e maynei' OR Acari* OR housedust* OR (dust NEAR/6 (allerg* OR sensiti* OR hypersensiti* OR indoor* OR house* OR domestic* OR asthma* OR ambient*))):ab,ti) AND ('air conditioning'/de OR 'exposure'/de OR 'dust exposure'/de OR 'environmental exposure'/de OR 'environmental parameters'/de OR 'avoidance behavior'/de OR 'environmental factor'/de OR 'environmental management'/de OR 'textile'/de OR 'home environment'/de OR 'tertiary prevention'/de OR 'microclimate'/de OR 'room ventilation'/de OR 'air quality'/de OR 'ambient air'/de OR 'air quality control'/de OR humidity/de OR 'environmental sanitation'/de OR 'sanitation'/de OR (avoidance* OR (impermeab* NEAR/3 cover*) OR ((humid* OR allergen* OR climate*) NEAR/3 (control* OR reduction*)) OR (air NEAR/3 (condition* OR filt* OR qualit* OR ambient* OR control* OR clean*)) OR ventilat* OR expos* OR textile* OR load OR environment* OR (dust NEAR/3 level*) OR anti-mite OR spray* OR mattress* OR management* OR (tertiary NEAR/3 prevent*) OR microclimate* OR micro-climate* OR sanitation OR bed-cloth* OR bed-cover* OR bedding OR furnish*):ab,ti) AND ('Controlled clinical trial'/exp OR 'Crossover procedure'/de OR 'Double-blind procedure'/de OR 'Single-blind procedure'/de OR (random* OR factorial* OR crossover* OR (cross NEXT/1 over*) OR placebo* OR ((doubl* OR singl*) NEXT/1 blind*) OR assign* OR allocat* OR volunteer* OR trial OR groups):ab,ti) NOT ([animals]/lim NOT [humans]/lim) NOT ([Conference Abstract]/lim) AND [English]/lim

List of included and excluded studies in the updated search.

	Author	Year	Included?	Rationale
1	Eick	2011	No	not patients with house dust mite-allergic asthma
2	Glasgow	2011	No	excluded by Gotzsche and Johansen
3	Maas	2011	No	not tertiary prevention
4	Neymayr	2011	No	not a clinical trial
5	Takaro	2011	No	not randomized
6	Breysse	2012	No	not a clinical trial
7	Celano	2012	No	not patients with house dust mite-allergic asthma
8	El-Ghitany	2012	Yes	
9	Gehring	2012	No	not tertiary prevention
10	Ho	2012	No	abstract
11	Masna	2012	No	abstract
12	Scott	2012	No	not tertiary prevention
13	NCT	2013	No	protocol issue
14	Tsurikisawa	2013	No	not blinded
15	Hogaard	2014	No	abstract
16	NCT	2014	No	duplicate
17	Hogaard	2014	No	duplicate
18	Murray	2015	No	duplicate
19	Smith	2015	No	not blinded
20	Sumner	2015	No	duplicate
21	Dimango	2016	No	not patients with house dust mite-allergic asthma
22	NCT	2016	No	protocol issue
23	Tsurikisawa	2016	No	not blinded
24	Winn	2016	No	not a clinical trial
25	Luo	2017	No	abstract
26	Murray	2017	Yes	
27	Morten	2018	No	not patients with house dust mite-allergic asthma
28	Bjermer	2019	No	not a clinical trial

Number of trials available per subgroup.

Category	FEV_1 %pred.	PC_{20}	Std. ASSs
Steroids	9	9	7
No steroids	5	5	4
Child	5	6	5
Adult	11	9	7
Co-sensitization	8	9	7
No co-sensitization	2	2	2

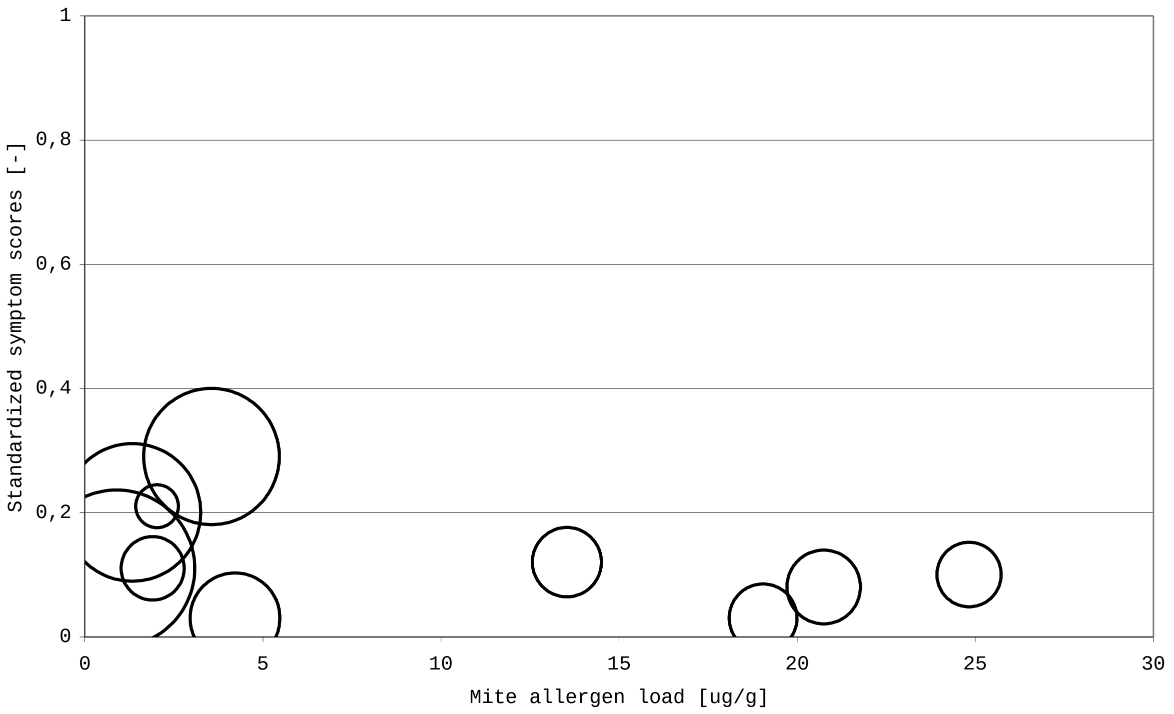

Figure. Bel plot for the standardized asthma symptom scores against the mite allergen load from the mattress at baseline.

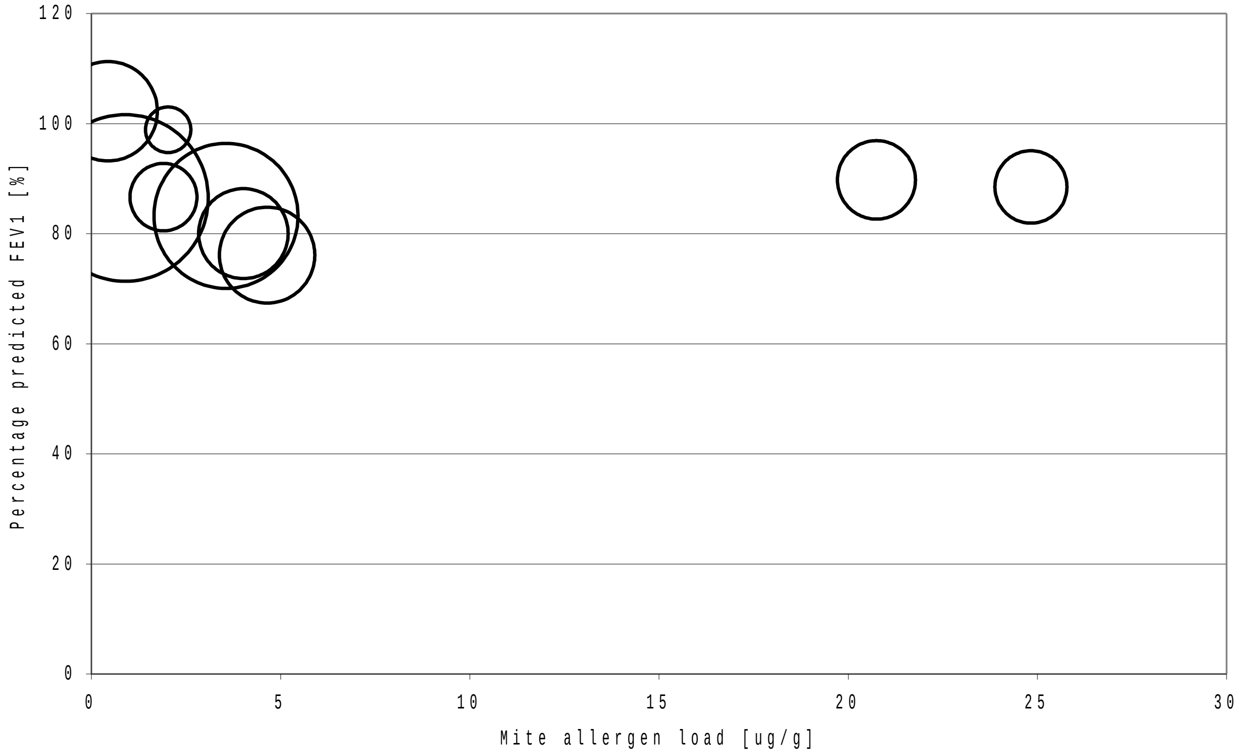

Figure. Bel plot for the FEV_1 percentage of predicted against the mite allergen load from the mattress at baseline.

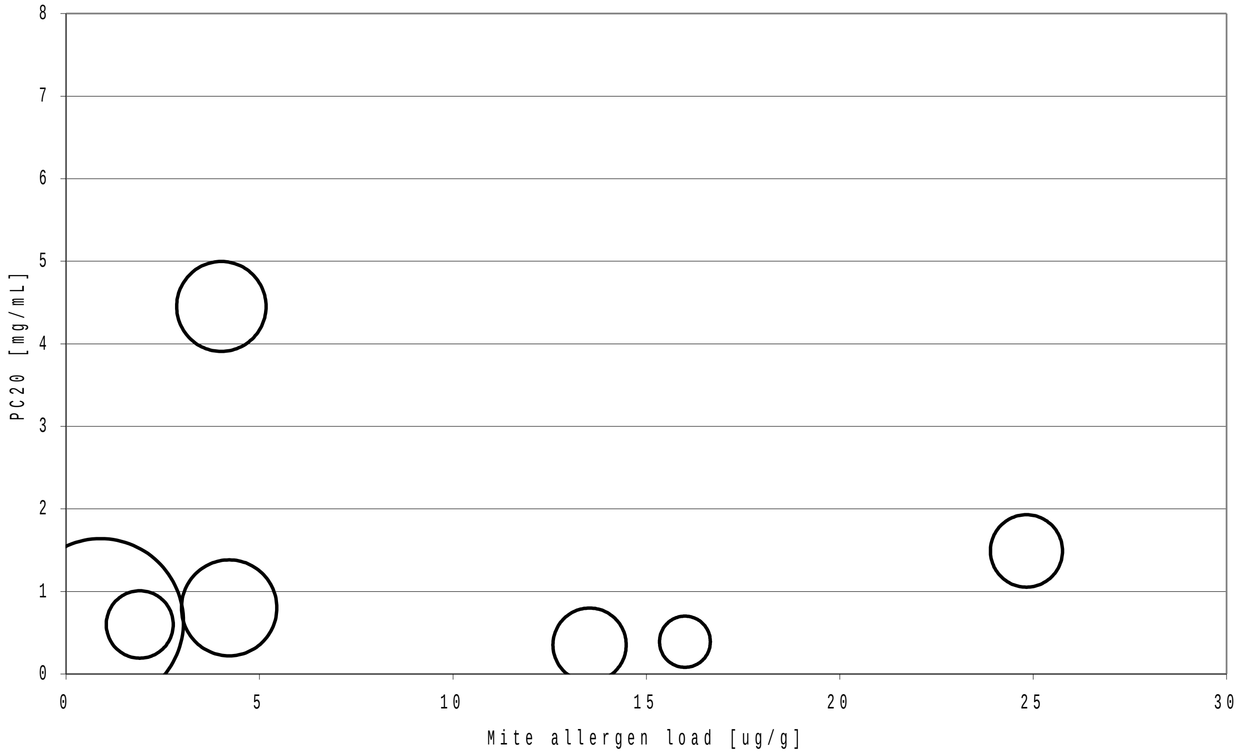

Figure. Bel plot for the PC_{20} against the mite allergen load from the mattress at baseline.
